# HIV-1 Tat Inhibits Autotaxin Lysophospholipase D Activity and Modulates Oligodendrocyte Differentiation

**DOI:** 10.1177/1759091416669618

**Published:** 2016-09-22

**Authors:** Natalie A. Wheeler, Babette Fuss, Pamela E. Knapp, ShiPing Zou

**Affiliations:** 1 Department of Anatomy and Neurobiology, Virginia Commonwealth University School of Medicine, Richmond, VA, USA; 2 Department of Pharmacology and Toxicology, Virginia Commonwealth University School of Medicine, Richmond, VA, USA; 3 Institute for Drug and Alcohol Studies, Virginia Commonwealth University School of Medicine, Richmond, VA, USA

**Keywords:** neuroAIDS, autotaxin, Tat, oligodendrocyte, lysophosphatidic acid, myelin

## Abstract

White matter injury has been frequently reported in HIV^+^ patients. Previous studies showed that HIV-1 Tat (transactivator of transcription), a viral protein that is produced and secreted by HIV-infected cells, is toxic to young, immature oligodendrocytes (OLGs). Adding Tat to the culture medium reduced the viability of immature OLGs, and the surviving OLGs exhibited reduced process networks. OLGs produce and secrete autotaxin (ATX), an ecto-enzyme containing a lysophospholipase D (lysoPLD) activity that converts lysophosphatidylcholine (LPC) to lysophosphatidic acid (LPA), a lipid signaling molecule that stimulates OLG differentiation. We hypothesized that Tat affects OLG development by interfering with the ATX-LPA signaling pathway. Our data show that Tat treatment leads to changes in the expression of OLG differentiation genes and the area of OLG process networks, both of which can be rescued by LPA. Tat-treated OLGs showed no change in LPA receptor expression but significantly decreased extracellular ATX levels and lysoPLD activity. In Tat transgenic mice, expression of Tat *in vivo* leads to decreased OLG ATX secretion. Furthermore, co-immunoprecipitation experiments revealed a potential physical interaction between Tat and ATX. Together, these data strongly suggest two functional implications of Tat blocking ATX’s lysoPLD activity. On one hand, it attenuates OLG differentiation, and on the other hand it interferes with the protective effects of LPA on OLG process morphology.

## Introduction

Neurocognitive complications are frequently reported in HIV^+^ patients. Although combined anti-retroviral therapy (cART) effectively blocks HIV replication in the central nervous system (CNS) and profoundly decreases the incidence of severe neurocognitive impairments, such as HIV-associated dementia, roughly 50% of the HIV-infected population still develop various forms of milder neurocognitive deficits, collectively called HIV-associated neurocognitive disorders (HAND; Ellis et al., 2007; McArthur et al., 2010). White matter injuries in HIV-infected patients are commonly observed and have been shown to correlate to HAND pathogenesis (Filippi et al., 2001; Archibald et al., 2004; Pfefferbaum et al., 2007). Although cART can reduce the viral load in the cerebrospinal fluid (CSF) to below detectable levels, white matter damage and HAND persists (Yilmaz et al., 2006). This implies that toxic factors released by previously infected CNS cells may inflict more damage than the virus itself.

One candidate factor for HIV-induced white matter injury is the HIV-1 viral protein Tat (transactivator of transcription), which is a key component for efficient transcription of the HIV genome. Tat has been shown to be continuously expressed and secreted by infected CNS cells and can be detected in the CSF (Wang et al., 2014) of HIV patients, even with cART (Johnson et al., 2013). Previously, we have reported that Tat significantly decreases immature OLG viability (Zou et al., 2015). Moreover, Tat exposure also reduced the process network formed by fine, branching processes that are typical at this stage, suggesting that Tat may also inhibit immature OLG differentiation.

OLG development has been extensively studied and well characterized both *in vivo* and *in vitro*. Newly formed oligodendroglial precursor cells (OPCs) migrate to their targeted destination to differentiate into pre-myelinating OLGs, which ultimately evolve into mature OLGs and myelinate axons. It is also well established that OLG differentiation is controlled by the fine-tuning of both intrinsic factors, such as transcriptional control and epigenetic regulation, and extracellular signals, including growth factors and mitogens (Zuchero and Barres, 2013; Mitew et al., 2014). One extracellular protein that has been shown to play a significant role in OLG development is autotaxin (ATX), also known as ENPP2, phosphodiesterase-Iα/ATX, or lysophospholipase D (lysoPLD). ATX is predominantly expressed and secreted by OLG lineage cells (Fox et al., 2003; Zhang et al., 2014) and has been shown to promote OLG development along the lineage via its two functional domains (Dennis et al., 2005; Wheeler et al., 2015). The first domain, the modulator of OLG remodeling and focal adhesion organization (MORFO), was found to promote the morphological maturation of OLGs (Dennis et al., 2008). The second domain, the enzymatic lysoPLD-active site, generates the lipid signaling molecule lysophosphatidic acid (LPA; Tokumura et al., 2002; Umezu-Goto et al., 2002; Nakanaga et al., 2010), which promotes OLG differentiation by epigenetically regulating the expression of OLG differentiation genes (Wheeler et al., 2015).

Since both Tat and ATX affect immature OLGs, we hypothesized that Tat inhibits OLG differentiation by interfering with the ATX-LPA signaling pathway. Our studies showed that 18 hr Tat treatment led to a reduction in OLG process network area and in expression of the OLG differentiation genes, *Ugt8* and *Cnp*. Although LPA by itself did not affect OLG morphology, it protected OLGs from Tat-induced process retraction, and rescued gene expression down-regulated by Tat. Tat also decreased both extracellular ATX level and ATX’s lysoPLD activity. In addition, co-immunoprecipitation (Co-IP) indicated a potential binding between Tat and ATX, which may account for the reduced ATX secretion and lysoPLD activity. Together, these results suggest that Tat may physically bind to ATX, inhibiting both ATX secretion from OLGs and the extracellular lysoPLD activity of ATX, and consequently, LPA signaling. Disrupting interactions between ATX and Tat may be a potential therapeutic strategy for protecting HIV patients from white matter injury.

## Materials and Methods

All animal procedures were performed strictly in compliance with protocols reviewed and approved by the Virginia Commonwealth University Institutional Animal Care and Use Committee (IACUC).

### Transgenic Mice

For *in vivo* Tat expression, a Tat-transgenic mouse line was used as previously described (Bruce-Keller et al., 2008). In brief, expression of Tat was under the control of a tetracycline responsive element (TRE), which initiates the transcription of the tat gene once it is bound by a protein complex formed by the reverse tetracycline transactivator (rtTA) and doxycycline (DOX). Both the Tat^+^ mice and their Tat^−^ littermates were genetically engineered to express rtTA under the glial fibrillary acidic protein promoter. As previously reported by our lab and others, Tat mRNA can be detected in the CNS of Tat^+^ mice within 48 hr DOX treatment (Bruce-Keller et al., 2008). Three-month-old Tat^+^ transgenic mice and their Tat^−^ siblings, regardless of sex, were fed with DOX-containing chow (6 g/kg) *ad libitum* for 10 days.

### Pharmacological Compounds

LPA (18:1; Sigma, St. Louis, MO) was dissolved in DMEM containing 0.1% fatty acid-free bovine serum albumin. Recombinant Tat_1–86_ and biotin-conjugated Tat (biotin-Tat, ImmunoDx, LLC Woburn, MA) were dissolved in distilled water. In the experiments involving pharmacological compounds, vehicle controls refer to an equal volume of the solvent that is used to dissolve the compounds.

### ATX-lysoPLD Activity Assay

ATX-lysoPLD activity was determined using a fluorogenic assay previously described (Ferguson et al., 2006; Wheeler et al., 2015). In brief, primary OLG cultures were grown in phenol-red-free media and treated with vehicle or Tat for 18 hr before supernatants were collected and concentrated (40×) via centrifugal filters (EMD Millipore, Billerica, MA). The concentrated supernatants were then incubated with 2.5 μM FS-3 substrate (Echelon Biosciences Inc., Salt Lake City, UT) at 37℃ for 4 hr. Additionally, to determine whether Tat directly decreases ATX lysoPLD activity, conditioned medium from untreated primary OLG cultures grown in phenol-red-free DMEM was concentrated (40×) before either vehicle or Tat (100 nM) and 2.5 μM FS-3 substrate were added (Echelon Biosciences Inc., Salt Lake City, UT) at 37℃ and incubated for 4 hr. Changes in fluorescence with time were measured at an excitation wavelength of 485 nm and an emission wavelength of 520 nm using a PHERAstar multimode microplate reader (BMG LABTECH Inc. Cary, NC.).

### RNA Isolation and Real-Time RT-qPCR Analysis

Isolation of RNA from control and treated OLGs (Tat ± LPA) was performed as previously published (Wheeler et al., 2015). The following unmodified mouse gene-specific primer pairs were used:

*Cnp:*

forward (5′-ATGCCCAACAGGATGTGGTG-3′),

reverse (5′-AGGGCTTGTCCAGGTCACTT-3′)

*Ugt8:*

forward (5′-AGGAGCTCTGGGGAGATTGC-3′),

reverse (5′-TTTGAATGGCCAAGCAGGTCA-3′)

*Atx:*

forward (5′-GACCCTAAAACCATTATTGCTAA-3′),

reverse (5′-GGGAAGGTGCTGTTTCATGT-3′)

*Lpar1:*

forward (5′-GCCACCTGGCTGCTGCAGA-3′),

reverse (5′-GTGTCGATGAGGCCCTGCCG-3′)

*Lpar2:*

forward (5′-CTGTTCAGCCGCTCCTACCTGG-3′),

reverse (5′-GGCAGCTGACGTGCTCTCTGCCATAG-3′)

*Lpar3:*

forward (5′-ACGAGCTTCGTCCCCGTCCA-3′),

reverse (5′-ACGAGCTTCGTCCCCGTCCA-3′)

*Lpar4:*

forward (5′-GCCTTGGTACGTTCCCAAGCCATT-3′),

reverse (5′-AGTTGCAAGGCACAAGGTAATCGGG-3′)

*Lpar5:*

forward (5′-GGGACTAGAGGGGAGCTCACCGAA-3′),

reverse (5′-TAGCCTCTGGCTGGTGGCATCCTAG-3′)

*Lpar6:*

forward (5′-GTAAGCGCCAACGGCTCCA-3′),

reverse (5′-GTAAGCGCCAACGGCTCCCA-3′)

*Pgk1: (as reference gene):*

forward (5′-ATGCAAAGACTGGCCAAGCTAC-3′),

reverse (5′-AGCCACAGCCTCAGCATATTC-3′)

RT-qPCR reactions with at least two technical replicates per sample were performed on a CFX96 real-time PCR detection system (BioRad, Hercules, CA) using the iQ SYBR Green Supermix (BioRad, Hercules, CA). PCR conditions were as follows: 95℃ for 3 min followed by 40 cycles of 95℃ for 15 s, 58℃ for 30 s, and 95℃ for 10 s. For all primer pairs, melting curves were used to ensure specificity. Relative expression levels were determined using the ΔΔCT method (Livak and Schmittgen, 2001).

### Western Blot

Concentration of proteins extracted from cultured OLGs was determined using a BCA protein assay kit (ThermoFisher Scientific, Waltham, MA). Equal amounts of protein samples were loaded on a Criterion precast 4% to 20% gradient SDS gel (Bio-Rad, Hercules, CA), electrophoretically separated, and probed with antibodies specific to 2′,3′-cyclic-nucleotide 3′-phosphodiesterase (CNPase; Abcam, Cambridge, MA). Bound primary antibodies were detected with appropriate IRDye secondary antibodies (1:3000, Li-COR, Lincoln, NE), and imaged using an Odyssey Imager (Li-COR). Protein bands (sum of CNP1 and CNP2) were quantified using Li-COR image studio software.

### Primary OLG Culture

Immature murine OLGs were cultured as previously described (Zou et al., 2015). In brief, primary mixed glial cells isolated from brains of postnatal day 0 to 2 mouse pups (CD-1, Charles River Laboratory, Wilmington, MA), irrespective of sex, were plated in Dulbecco’s Modified Eagle’s Medium (DMEM; Life Technologies, Carlsbad, CA) supplied with fetal bovine serum (10%, Thermo Scientific Hyclone, Logan, UT), glucose (6 g/L, Sigma, St. Louis, MO), sodium bicarbonate (0.13%, Life Technologies), and penicillin/streptomycin (1×, Life Technologies). Culture medium was refreshed every other day. At day 8, O2A/glial progenitor cells were collected, as previously described (Zou et al., 2015), from the mixed cultures and replated on poly-L-lysine coated 12-well plates or cover slips, at a density of 250,000 cells per well or 15,000 cells per cover slip, and incubated in DMEM supplied with CNTF (10 ng/ml, Peprotech, Rocky Hill, NJ), N-Acetyl Cystein (NAC; 5 μg/ml, Sigma) and triiodothyronine (15 nM, Sigma) for 2 days to reach immature status before further experiments.

### Time-Lapse Imaging

Time-lapse imaging of individual OLGs was performed as described by (Zou et al., 2011). In brief, OLGs were cultured in 12-well plates for 2 days before treatment with vehicle or Tat. Culture plates were then transferred to the environmental chamber (37℃, 5% CO_2_) of a Zeiss Axio Observer Z1 system (Carl Zeiss Microscopy, LLC, Thornwood, NY). Individual OLGs were randomly selected and imaged hourly for 18 hr then blindly analyzed using the Zeiss Axiovision 4.8 software.

### Immunocytochemistry and Image Analysis

O4 immunostaining was performed on live, unfixed cells. All other immunostaining was performed after cells were fixed with 4% paraformaldehyde. O4 monoclonal antibodies (1:20, grown from hybridoma cells in our lab; Knapp and Hauser, 1996) were applied at room temperature for 15 min. Goat anti-mouse IgM-Cy3 (1:1000, Millipore, Billerica, MA) was used to probe bound O4 antibodies. All LPA receptor antibodies (Anti- LPAR1 (Abcam), LPAR2, and LPAR3; Santa Cruz, Dallas, TX; LPAR4, Alomone labs, Hadassah Ein Kerem, Israel; and LPAR6, Acris, San Diego, CA) were applied at 1:1000 at room temperature for 1 hr, and corresponding secondary antibodies were conjugated to Alexa 488 (1:2000, Life Technologies). Antibody specific to ATX (Cosmo Bio, Japan) was also used at 1:1000 at room temperature. The cell nucleus was stained with Hoechst 33342 dye (1:2000, Life Technologies). Images were taken on a confocal microscope (Zeiss LSM 700, Carl Zeiss, Thornwood, NY) and processed using Zeiss Zen 2010 software. OLG morphology was assessed by determining the overall process network (total O4^+^ area minus the area occupied by the cell body) as described previously (Dennis et al., 2008), but using ImageJ software (NIH). Evaluators were blinded to treatment groups.

### Immunohistochemistry

Three-month old Tat^+^ and Tat^−^ mice, both female and male, were perfused with 4% paraformaldehyde after they were fed with DOX-containing chow for 10 days. The perfused brain was dissected out and fixed with 4% paraformaldehyde overnight before washing three times in PBS and immersed in 20% sucrose solution for 24 hr. The brains were then frozen in OCT compound (Sakura Finetek, Torrance, CA), coronally sectioned at 20 µm thickness, and thaw-mounted onto Superfrost-plus slides (Fisher). The sections were then immersed in acetone at −20℃ for 20 min before they were washed with PBS and blocked with PBS containing 0.5% Triton and 5% cold fish gelatin (Electron Microscopy Science, Hatfield, PA) for 15 min at room temperature and probed with Rb anti-ATX (1:250, Cosmo Bio, Japan) and Ms anti-APC (1:100, Millipore) overnight at 4℃. Images were taken on a confocal microscope (Zeiss LSM 700, Carl Zeiss, Thornwood, NY) and processed using Zeiss Zen 2010 software.

### Co-Immunoprecipitation

Dynabeads Protein G (1.5 mg/50 μl, Life Technologies) was first mixed with primary antibodies (5 µg): non-specific mouse IgG (Abcam), mouse anti-V5 (Abcam), rabbit anti-biotin (Abcam), or rabbit anti-ATX (Cayman Chemical, Ann Arbor, MI) and washed three times with PBS at room temperature. Concentrated supernatant (500 μl) from a COS-7 cell line or primary OLG culture was mixed with 5 µg biotin-Tat and added to the Dynabeads-bound primary antibodies and gently rocked overnight at 4℃. The next day, Dynabeads (with bound proteins) were washed three times at room temperature with PBS before being collected and then resuspended in loading buffer (2× Laemmli buffer, Bio-Rad), heat denatured (95℃, 5 min) and loaded (40 µl/sample) to a 4% to 20% SDS-PAGE precast gel (Bio-Rad) for Western blotting.

### Statistical Analysis

OLG process network and Western blot of CNP were analyzed using one-way ANOVA followed by post hoc Bonferroni’s testing (Figure 2(b) and 3(b)). Gene expression (Figure 3(a), (c) and (b)) data obtained by RT-PCR were analyzed using one-sample *t* test. Western blot of intracellular and extracellular ATX in cultured OLGs (Figure 5(b)), and quantification of ATX^+^CC1^+^ cells and extracellular ATX^+^ pixels in Tat transgenic mouse brain sections (Figure 6(b) and (c)), were analyzed using student’s *t* test. Autotaxin lysoPLD activity assay was analyzed using two-way ANOVA (treatment, time) followed by post hoc Bonferroni’s testing (Figures 5(c), (d), 6(a), and 7(d)). For all statistics, a *p* value ≤ .05 was considered significant.

**Figure 1.**
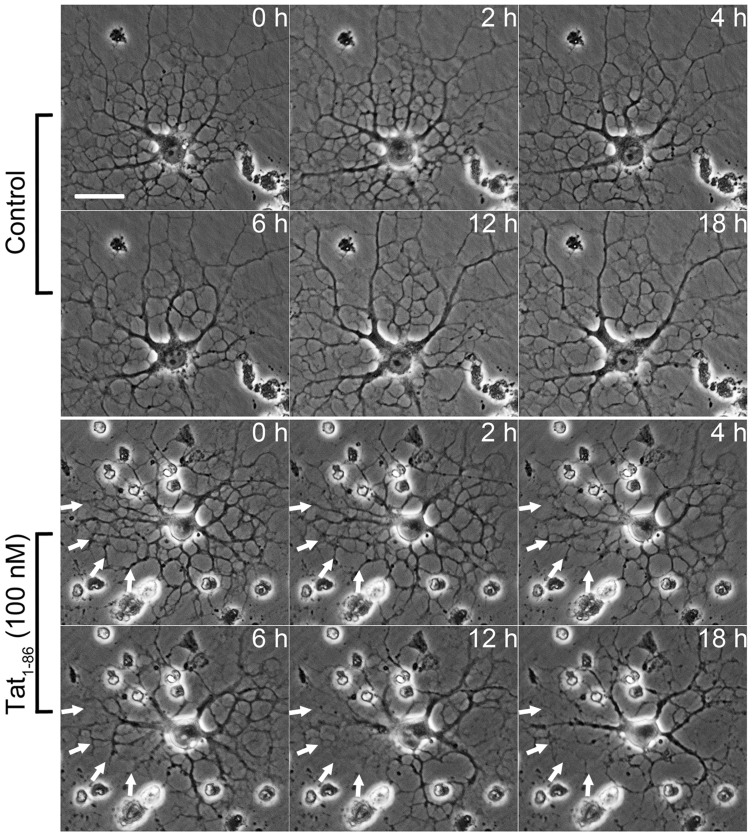
HIV-1 Tat decreases immature OLG process networks. Sample time-lapse images of immature OLGs treated with vehicle− or 100 nM Tat at 0, 2, 4, 6, 12, and 18 hr. Tat treatment leads to decreased process network, especially after 4 hr, while process networks are stable or growing in vehicle-treated OLGs. (Arrows; Scale bar = 20 µm).

**Figure 2.**
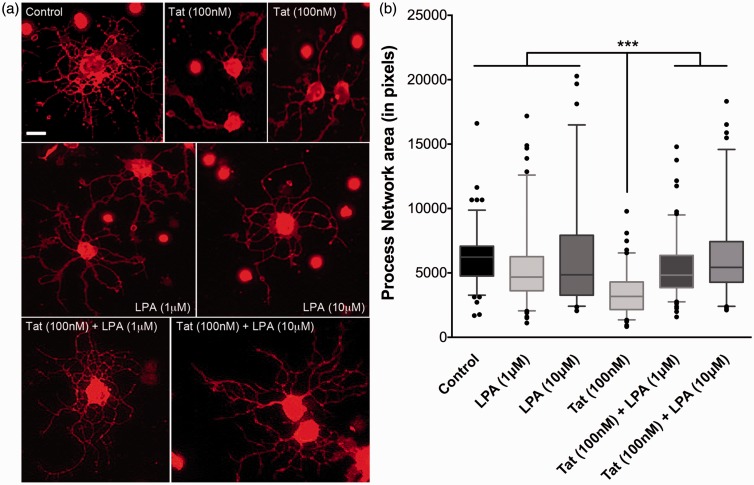
LPA reverses the Tat-induced decrease in immature OLG process networks. (a) Representative images of Vehicle-(control), Tat-, LPA-, and Tat+LPA -treated OLGs shown by O4+ immunostaining (Scale bar = 10 µm). (b) Tat significantly decreased OLG process networks at 18 hr. LPA at 1 or 10 μM rescued the decreased OLG process network induced by 100 nM Tat. LPA by itself at both concentrations has no effect on OLG process networks. (***p < .001 vs. 100 nM Tat; one-way ANOVA followed by post hoc Bonferroni’s test, *N* = 4 independent experiments, ≥30 cells were counted for each *N*).

**Figure 3.**
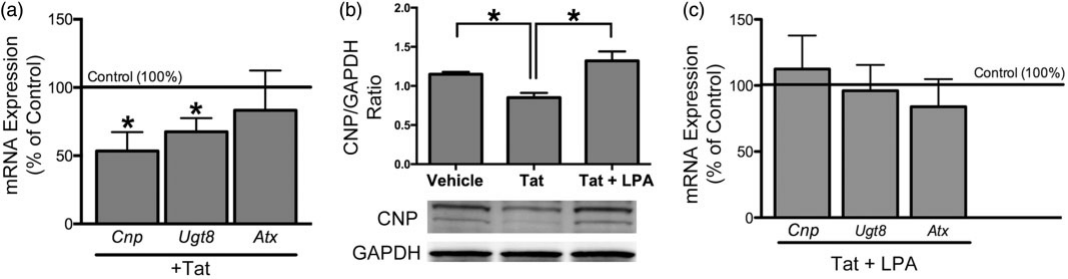
OLG differentiation genes are downregulated with Tat treatment, an effect that is rescued by LPA. (a) qRT-PCR showed that 18 hr Tat exposure decreased expression of *Ugt8* and *Cnp* but had no effect on expression of *Atx*. (b) Western blot showed that 18 hr Tat treatment decreased CNP levels in immature OLGs, which was also reversed by LPA. (c) qRT-PCR showed that Tat-induced decrease of *Ugt8* and *Cnp* expression can be rescued by adding LPA to medium. Concurrent addition of Tat and LPA also had no effect on *Atx* expression. (b: One-way ANOVA followed by post hoc Bonferroni’s testing; a, c: One sample *t* test; *p < .05 vs. vehicle; *N* = 4 independent experiments).

**Figure 4.**
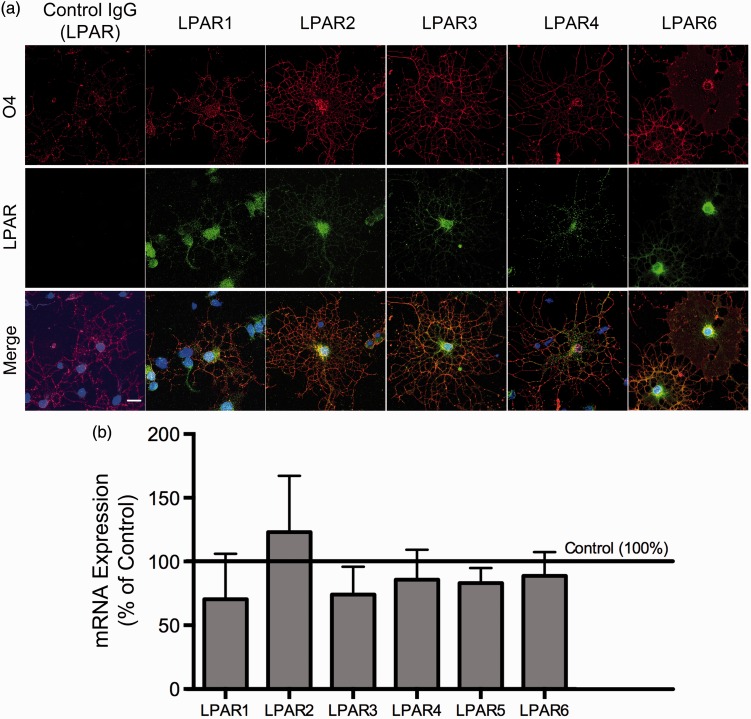
Expression of LPA receptors is not affected by Tat treatment. (a) Immunostaining detects LPAR1–4 and 6 on O4^+^ immature OLGs (Scale bar = 10 µm). (b) qRT-PCR gene expression data showing LPAR1–6 mRNA in OLGs is not altered by Tat treatment. The mean value for vehicle-treated OLGs was set to 100% (horizontal line), and values for Tat-treated cells were calculated accordingly. (One sample *t* test; *N* = 4 independent experiments).

**Figure 5.**
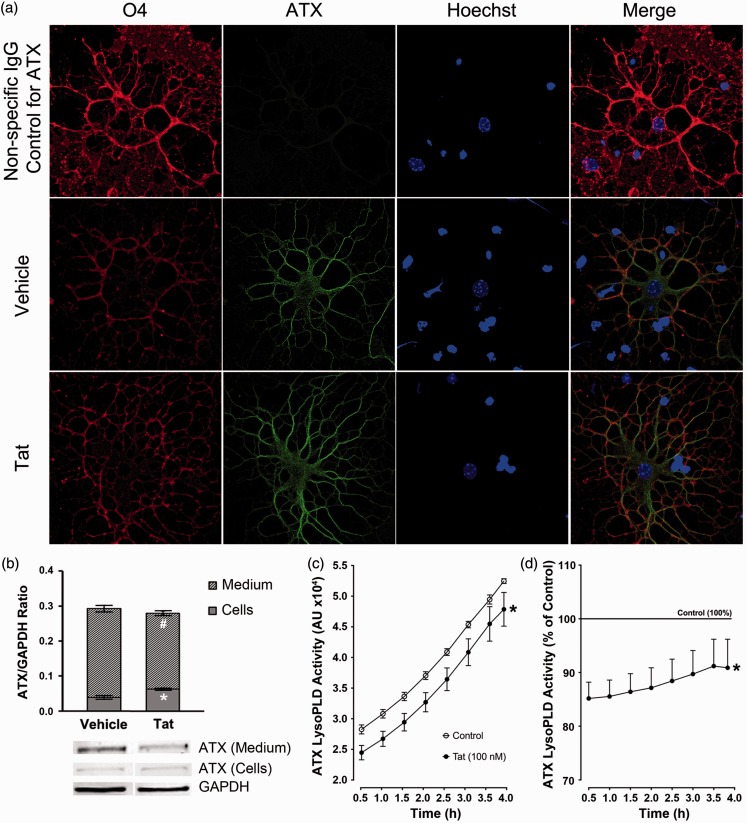
Tat inhibits ATX secretion and decreases ATX lysoPLD activity *in vitro*. (a) Immunostaining depicting intracellular ATX in Tat- or vehicle-treated OLGs. (b) Western blot results suggest that the amount of ATX in the medium (extracellular ATX) in Tat-treated OLG cultures is significantly less than in vehicle-treated OLG cultures. On the other hand, the intracellular ATX in Tat-treated OLGs is higher than in vehicle-treated OLGs. (c) Tat treatment significantly decreases overall lysoPLD activity in the medium. (d) The mean value of the lysoPLD activity of supernatant from vehicle-treated OLGs was set to 100% (horizontal line). Tat treatment leads to 10% to 15% decrease of lysoPLD activity at all time points examined (b: *,^#^*p* ≤ .05, vs. vehicle treatment, student’s *t* test; c, d: *p < .05 vs. vehicle; main effect, two-way ANOVA (Tat, Time) followed by post hoc Bonferroni’s test, *N* = 5 independent experiments).

**Figure 6.**
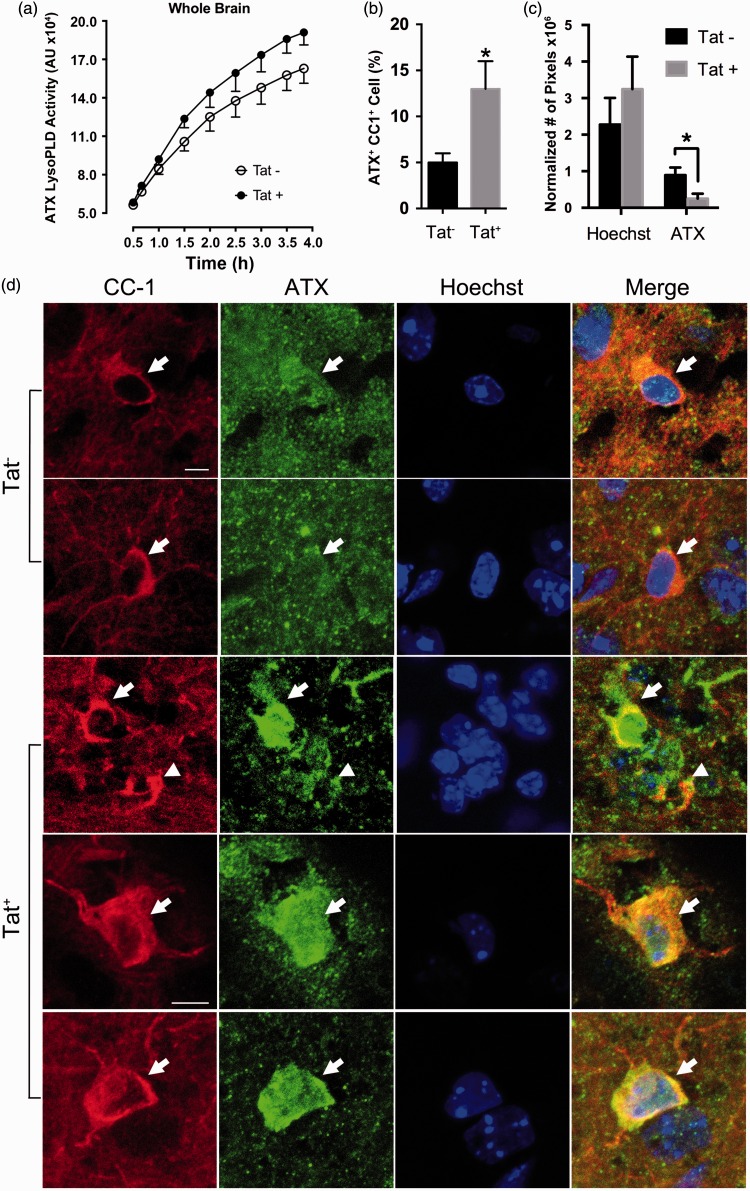
Expression of Tat *in vivo* inhibits OLG ATX secretion. (a) ATX lysoPLD activity assay using whole brain homogenate showed that CNS expression of Tat did not change overall ATX lysoPLD activities. (b) *in vivo* expression of Tat leads to increased amount of CC1^+^/ATX^+^ cells, indicating inhibition of ATX secretion from OLGs. (c) The amount of extracellular ATX in Tat^+^ or Tat^−^ brain slices were quantified by counting ATX^+^ pixels (green) that were not concurrently CC1^+^ (red) or Hoechst^+^ (blue, nuclear). Tat expression leads to significantly less ATX^+^/CC1^−^ pixels, while Hoechst^+^ pixels were not affected, indicating less extracellular ATX in Tat^+^ mice. (d) Sample IHC images depicting higher intracellular ATX levels in Tat^+^ mice (bottom three rows), when compared with Tat^−^ mice (top two rows). (Scale bar = 10 µm; a: two-way ANOVA (Tat, Time) followed by post hoc Bonferroni’s test; b,c: student’s *t* test; *p < .05 vs. control, *N* = 5 independent experiments).

**Figure 7.**
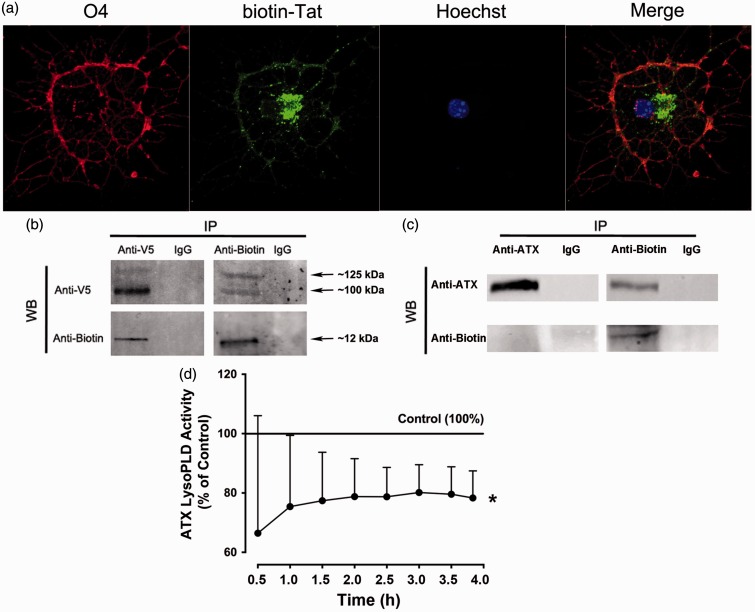
Potential physical interaction of Tat and ATX. (a) biotin-Tat was added to OLG culture media for 1 hr before immunostaining was performed. Fluorescent microscopic images demonstrated that the majority of biotin-Tat was in the OLG cytoplasm and processes. (b) Concentrated supernatant collected from a stably transfected Cos7 cell line that secretes V5-tagged ATX was mixed with biotin-Tat, and immunoprecipitated (IP) with antibodies specific to V5 (left panels) or biotin (right panels) and processed for Western blot (WB) for biotin (bottom panels) or V5 (top panels), respectively. Mouse IgG with no known specificity was used as negative control. While nothing was detected from msIgG precipitates, V5-tagged ATX was detected in anti-biotin precipitates, and vice versa. (c) Co-IP was also performed using biotin-Tat and concentrated supernatant collected from primary OLG culture. ATX can be detected in anti-biotin precipitates, but biotin-Tat can not be detected in anti-ATX precipitates. (d) Adding Tat to the supernatant collected from untreated primary OLG cultures significantly decreased the ATX lysoPLD activity when compared with vehicle control (*p < .05, vs. Control; two-way ANOVA (Tat, Time) followed by post hoc Bonferroni’s test, *N* = 4 independent experiments).

## Results

### Tat-Induced Decrease in Immature OLG Process Networks Is Reversed by LPA

Immature OLGs exposed to HIV-1 Tat show significantly decreased viability *in vitro* (Zou et al., 2015). In addition, surviving cells exhibit abnormal morphology, indicating that the differentiation of these cells is disrupted. To investigate the effect of Tat on the morphology of differentiating OLGs in more detail, immature OLGs were treated with vehicle or Tat for 18 hr before morphological analysis was performed. OLGs showed loss of fine, branching processes starting as early as 4 hr following Tat treatment, and ended up with significantly reduced process networks after the 18 hr experimental period (Figure 1).

Previous studies by Nogaroli et al. (2009) have shown that the addition of LPA to maturing OLGs increased membrane formation, and thus we examined whether LPA could reverse Tat-induced reduction of the OLG process network. Although the addition of 1 or 10 μM LPA seems to promote OLG differentiation (Figure 2(a)), it does not affect the total process network area of immature OLGs (Figure 2(b)). However, when added concurrently with Tat, LPA protected cells against Tat-induced process retraction.

### Tat-Induced Reduction of OLG Differentiation Gene Expression Can Be Reversed by LPA

We next investigated whether Tat also affects the expression of genes related to OLG differentiation such as, *Cnp* (2′,3′-cyclic nucleotide 3′-phosphodiesterase) and *Ugt8* (UDP-galactose ceramid galactosyltransferase 8). CNP expression is an early marker of OLG differentiation, and *Ugt8* is responsible for the synthesis of galactosylceramide, the major myelin glycosphingolipid (Kapitonov and Yu, 1997; Sprong et al., 1998; Marcus and Popko, 2002). An 18 hr Tat treatment significantly decreased the expression of both *Cnp* and *Ugt8* (Figure 3(a)). Since LPA protects against the negative effects of Tat on immature OLG process network area (Figure 2), we further examined whether LPA rescues the reduced gene expression in Tat-treated OLGs. Adding LPA concurrently with Tat completely rescued the expression of *Cnp* and *Ugt8* (Figure 3(c))*.* Additionally, CNP protein levels reduced by Tat treatment could be rescued by the addition of LPA (Figure 3(b)). OLG expression of *Cnp* and *Ugt8* has been reported to be regulated by the lysoPLD activity of ATX (Wheeler et al., 2015). However, qRT-PCR analysis showed that *Atx* expression itself is in OLGs not affected by either Tat or LPA (Figure 3(a) and (c)).

### Expression of LPA Receptors Is Not Affected by Tat Treatment

Since the effects of Tat on immature OLG morphology and differentiation gene expression can be rescued by LPA, we hypothesized that Tat interferes with the LPA signaling pathway. To assess the LPA receptor (LPAR) expression profile in our system, we performed immunostaining with antibodies specific to different LPARs in immature OLGs. Results revealed the presence of LPARs 1–4 and 6 on O4^+^ immature OLGs (Figure 4(a)). Due to a lack of an effective LPAR5 antibody, we can not verify the presence of LPAR5 in our OLG culture. However, LPAR5 mRNA was detected (Figure 4(b)). Furthermore, qRT-PCR performed on 18 hr Tat- or vehicle-treated OLGs showed that Tat treatment had no effect on LPAR 1–6 gene expression on immature OLGs (Figure 4(b)).

### Tat Decreases OLG Secretion of ATX and ATX lysoPLD Activity *in vitro*

Since LPAR expression was not affected by 18 hr Tat exposure, we next investigated whether Tat regulates ATX production. Although ATX is a secreted protein, immunostaining experiments showed that both vehicle- and Tat-treated OLGs are ATX positive, suggesting that a portion of ATX is retained in the cytoplasm, which could represent the pre-pro-enzyme population of ATX (Jansen et al., 2005; Figure 5(a)). Further studies by Western blot showed that the total amount of ATX, collected from both OLG culture media (secreted) and cells (intracellular), was not altered by Tat treatment. However, Tat treatment altered the distribution of ATX by significantly increasing intracellular ATX and lowering the level of extracellular ATX (secreted ATX) when compared with vehicle treatment (Figure 5(b)). Furthermore, media collected from OLG cultures treated with Tat exhibited significantly decreased lysoPLD activity compared with vehicle-treated cultures (Figure 5(c)), consistent with reduced ATX in the media. The reduction of lysoPLD activity in Tat-treated OLG media led to a ∼10% overall inhibition across all assay time points (Figure 5(d)).

### Expression of Tat *in vivo* Inhibits OLG ATX Secretion

The finding that Tat decreases extracellular ATX levels and lysoPLD activity *in vitro* led to further investigation of Tat’s effects *in vivo*. Protein extracted from the whole brain of Tat transgenic mice was used to assess the ATX lysoPLD activity *in vivo*. There was no significant difference in ATX lysoPLD activity between Tat^+^ and Tat^−^ mice (Figure 6(a)), suggesting that Tat expression does not affect overall ATX expression and/or activity *in vivo*. Furthermore, immunohistochemistry experiments showed significantly increased occurrence of cells that are both CC1^+^ and ATX^+^ (Figure 6(b) and (d)) in the brain of Tat^+^ mice, indicating increased amount of cytoplasmic ATX in OLGs. Additionally, the amount of extracellular ATX, quantified by counting ATX^+^ pixels (green) that were not concurrently CC1^+^ (red) or Hoechst^+^ (blue, nuclear), was significantly reduced in Tat^+^ mice (Figure 6(c)). Together, these data suggest that expression of Tat *in vivo* affects OLG ATX secretion

### Potential Physical Interaction Between Tat and ATX Decreases ATX Secretion

It has been well established that Tat can cross the plasma membrane and enter the cytoplasm of several different cell types (Frankel and Pabo, 1988; Mann and Frankel, 1991; Fawell et al., 1994). Hence, the finding that Tat reduces the rate of ATX secretion from OLGs, both *in vivo* and *in vitro*, prompted the hypothesis that Tat may interact with cytoplasmic ATX, and inhibits its secretion. We first verified the ability of Tat to enter the cytoplasm of OLGs. Images taken by confocal microscopy showed cytoplasmic localization of biotin-conjugated Tat within 1 hr after Tat was added to the medium (Figure 7(a)). To assess whether there exists a potential physical interaction between Tat and ATX, we performed Co-IP using biotin-Tat and V5-tagged ATX, collected and concentrated from the supernatant of a stably-transfected COS-7 cell line (Dennis et al., 2012). As seen in Figure 7(b), V5-tagged ATX was detected in anti-biotin precipitates and vice versa. As a control for non-specific binding, neither V5-tagged ATX nor biotin-Tat was detected in precipitates collected using a mouse IgG1 monoclonal isotype control (Figure 7(b)). It has been previously reported that ATX is synthesized as a pre-pro-enzyme, whose N-term 27 amino acid residues are proteolytically removed before secretion (Jansen et al., 2005). Since we used cell lines cultured in high density to harvest supernatants with high ATX concentration, and the antibody we used targets the C-term NUC domain of ATX, the two bands with different molecular weight (∼100 kDa and ∼125 kDa) seen in Figure 7(b) likely represent both the pre-pro-enzyme and the secreted ATX. To further verify the association between ATX and Tat in OLGs, the same Co-IP procedure was performed using biotin-Tat and concentrated supernatant collected from primary mouse OLG cultures. ATX was detected in anti-biotin precipitates (Figure 7(c)), indicating the existence of a potential physical interaction between ATX and Tat. Interestingly, in the same experiment, biotin-Tat was not detected in anti-ATX precipitates (Figure 7(c)), suggesting this interaction may be disrupted by the binding between ATX and the anti-ATX antibody. Additionally, when Tat or vehicle was added to the supernatant collected from untreated primary OLG cultures, Tat significantly decreased the lysoPLD activity in the supernatant (Figure 7(d)). Since this experimental paradigm bypassed any potential effect on cells, this result suggests that Tat directly interrupts the ATX lysoPLD activity, possibly due to its binding to ATX.

## Discussion

White matter injuries are frequently reported in HIV patients at all stages (Gongvatana et al., 2009; Zhu et al., 2013; Ragin et al., 2015). The mammalian adult CNS has been known to maintain a population of OLG precursor cells, which migrate to lesion sites upon injury and differentiate into myelinating OLGs to replace injured OLGs and regenerate lost, damaged myelin (Polito and Reynolds, 2005; Kang et al., 2010; Zuchero and Barres, 2013). Interestingly, white matter injury persists when HIV RNA is undetectable in the CSF (Yilmaz et al., 2006; Ragin et al., 2015). Our studies presented here show that HIV-1 Tat, which is continuously secreted by infected CNS cells, with or without viral replication, may physically interact with ATX. This interaction inhibits the secretion of ATX from OLGs and decreases extracellular ATX lysoPLD activity and subsequently, LPA production. Since the Tat-ATX interaction not only attenuates OLG differentiation, but also disrupts the protective effects of LPA on OLG process morphology, targeting this interaction may be a therapeutic strategy to promote recovery of white matter injury in HIV patients.

OLG lineage cells are targets of HIV-1 Tat. Proliferation and migration of OLG precursor cells, viability of immature OLGs, morphology, and myelin-like membrane production of mature OLGs have all been reported to be disrupted by Tat (Hauser et al., 2009; Hahn et al., 2012; Zou et al., 2015). Our previous studies using doxycycline-inducible Tat transgenic mice demonstrated that *in vivo* expression of HIV-1 Tat for 7 days leads to significantly increased active caspase-3 expression in O4^+^ OLGs (Hauser et al., 2009). Follow-up studies by Zou et al. (2015) further showed that 3-month expression of HIV-1 Tat in the CNS causes significantly decreased levels of myelin proteins MBP and MAG, and increased occurrence of abnormal myelin structures. In this study, our initial experiments found that immature OLGs that survived 18 hr Tat treatment exhibit a decrease in process network area (Figure 1). It is also plausible that Tat alters the viability of these cells at this stage represented by process retraction (Zou et al., 2015). Although LPA by itself does not promote process outgrowth, it protects against Tat-induced reduction of the OLG processes network area (Figure 2).

Previously, we have reported that early OLG differentiation is regulated by gene expression changes downstream of the ATX-LPA signaling axis (Wheeler et al., 2015). In this study, we found that Tat down-regulates OLG differentiation genes, *Utg8* and *Cnp* (Figure 3), which can be rescued by the addition of LPA (Figure 3), strongly supporting the hypothesis that Tat affects OLG differentiation by interfering with the LPA signaling pathway. Additionally, *Atx* gene expression in OLGs treated with Tat or LPA was not significantly different from control (Figure 3), indicating that the LPA rescue of *Cnp* and *Ugt8* gene expression was not attributed to altered *Atx* expression.

Since OLG *Atx* expression was not affected by Tat, we next assessed whether Tat changes the expression of LPA receptors. There are six *bona fide* LPARs, all of which are found on mice OLGs, with LPAR1 being the most abundantly expressed (Zhang et al., 2014). Our experiments showed that 18 hr Tat exposure did not affect the expression of any of the known LPAR genes in OLGs. Thus, it is highly probable that the deteriorating effects of Tat on OLGs are mediated via altered LPA production, as a result of decreased ATX lysoPLD activity, or an inhibition in the secretion of ATX from OLGs, rather than LPAR expression.

Tat did not affect overall OLG *Atx* gene expression or overall abundance of ATX. However, our Western blot results demonstrated a redistribution of ATX localization, with a lower amount of extracellular ATX, and a significantly higher amount of intracellular ATX in OLG cultures exposed to Tat (Figure 5(b)). This suggests that Tat may inhibit OLG ATX secretion. Accordingly, Tat-treated OLGs showed decreased extracellular ATX lysoPLD activity (Figure 5(c)). The extracellular ATX lysoPLD activity converts lysophosphatidylcholine (LPC) to LPA (Aoki et al., 2008), which has been shown to be the predominant source of extracellular LPA. Since LPA is needed for proper OLG development, reduced ATX lysoPLD activity may result in decreased LPA production, thus, leading to downregulated OLG differentiation gene expression. Hence, these results suggest a novel role for Tat in limiting the secretion of ATX from OLGs, thus reducing extracellular LPA production.

ATX lysoPLD activity was found unchanged in whole brain homogenates of Tat^+^ mice (Figure 6(a)). These protein samples, however, could not distinguish intracellular and extracellular ATX, and these data are thus in line with our *in vitro* findings, which showed that Tat treatment does not change overall OLG ATX expression. Furthermore, and consistent with our *in vitro* results, immunohistochemistry experiments showed that CNS expression of Tat significantly increased the percentage of ATX^+^CC1^+^ cells and decreased extracellular ATX level (Figure 6(b)–(d)), suggesting that Tat reduces the secretion of ATX from OLGs, and thus limits extracellular ATX levels.

It has been reported that HIV-1 Tat contains a membrane-penetrating sequence (amino acids 48–57), which enables it to translocate across the plasma membrane of various cell types (Mann and Frankel, 1991); however, it has yet to be reported that Tat can enter the cytoplasm of OLGs. Due to the relative low affinity of most Tat antibodies, we used biotin-Tat to assess whether Tat enters OLGs and affects ATX secretion. Immunocytochemistry and confocal microscopy showed that biotin-Tat was found clustered in the cytoplasm and major processes of OLGs within 1 hr after adding to the culture media (Figure 7(a)). Both Tat and ATX have been reported to physically interact with other cellular components such as integrin receptors (Hausmann et al., 2011; Urbinati et al., 2012), indicating the possibility of a protein complex formed by Tat and ATX and other cellular components. Alternatively and more speculatively, the crystal structures of Tat and ATX reveal that both proteins contain cysteine-rich domains (Tahirov et al., 2010; Nishimasu et al., 2011; Gu et al., 2014), indicating the potential of forming intermolecular disulfide bonds. We thus hypothesized that cytoplasmic Tat may bind ATX and limit its secretion rate. Co-IP showed that biotin-Tat and V5-tagged ATX precipitated together (Figure 7(b)), indicating a potential physical interaction between Tat and ATX. Since the V5-tagged ATX used in the Co-IP was collected from the supernatant of a stably transfected cell line, the concentration of ATX cannot be effectively determined and may exceed the physiological concentration of ATX *in vivo*. To further validate this interaction between Tat and ATX in OLGs, Co-IP was also performed using ATX collected from supernatant of primary OLG culture and biotin-Tat. In this setting, we found that ATX can be precipitated by biotin-Tat but not vice versa (Figure 7(c)). The anti-ATX antibody we used in this study recognizes and binds the C-terminal fragments of ATX. It is possible that this binding may alter the conformation of ATX, which disrupts the ATX-Tat interaction. In this case, altered ATX conformation may also lead to altered lysoPLD activity. Consistently, we found that Tat significantly decreased the ATX lysoPLD activity when added directly to the supernatant collected from untreated OLG culture (Figure 7(d)). Another possibility is that the Tat binding site on ATX overlaps the antibody binding site, and thus was shielded by the bound antibody. Further studies are required to better characterize the binding site, affinity and stoichiometry of the ATX-Tat interaction, as well as whether this interaction requires additional adaptor proteins.

**Figure 8.**
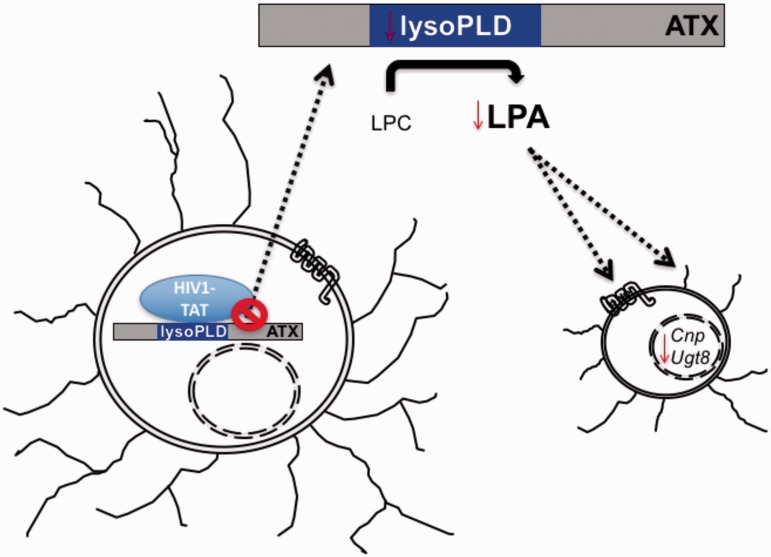
Proposed model for effects of Tat on immature OLG differentiation. In the CNS of HIV^+^ patients, extracellular Tat, produced by productively infected cells such as microglia, enters the OLG cytoplasm and binds to ATX. This interaction between Tat and ATX may impede ATX secretion and lead to reduced levels of extracellular ATX, and downregulated expression of OLG differentiation genes such as *Ugt8* and *Cnp*.

Taken together, the studies reported here suggest an injury mechanism where HIV-1 Tat expressed by infected CNS cells in HIV^+^ patients enters the cytoplasm of neighboring OLGs, binds cytoplasmic ATX, and inhibits OLG ATX secretion. Limited ATX secretion by OLGs reduces the extracellular concentration of ATX and thereby reduces its overall ATX lysoPLD activity and the production of LPA, which consequently down-regulates OLG differentiation gene expression and compromises the protective role of LPA against Tat-induced OLG injury (Figure 8).
